# Takotsubo cardiomyopathy – stunning views on the broken heart

**DOI:** 10.1007/s12471-016-0869-8

**Published:** 2016-07-15

**Authors:** A. J. Teske, J. W. Verjans

**Affiliations:** University Medical Center Utrecht, Utrecht, The Netherlands

Takotsubo cardiomyopathy is a poorly understood myocardial disorder that is generally considered to be a transient, non-ischaemic disease with a favourable prognosis in post-menopausal women. Its profile is typically characterised by cardiomyopathy involving regional wall motion abnormalities after a stressful event, but is also associated with serious life-threatening complications in up to 10 % of cases [1].

While this disease entity has received a large amount of attention over the past years, the aetiology still remains an enigma. Although it has been postulated that catecholamine and endothelin excess likely play a central role, the exact pathophysiology is poorly understood. The triggering event is typically diverse, ranging from severe emotional stress and even pleasant emotional stressors, but often no trigger can be identified [2].

The diagnosis of takotsubo cardiomyopathy is challenging, and can only be made after significant coronary artery disease has been ruled out. Only modestly raised troponins and often a vastly raised B‑type natriuretic peptide are often seen. Finally, this disease presents in many shades of grey, the typical apical ballooning, but also mid-ventricular, basal, and focal subtypes have been described (for different clinical examples see http://specklepedia.com/examples/miscellaneous/tako-tsubo-cardiomyopathy/).

To further elucidate this still relatively unknown disease, the current issue of the* Netherlands Heart Journal* features three unique articles from different research groups, each addressing a specific and unrelated topic associated with the same disease. This work will likely further help us grasp this heterogeneous disease.

**Fig. 1:**
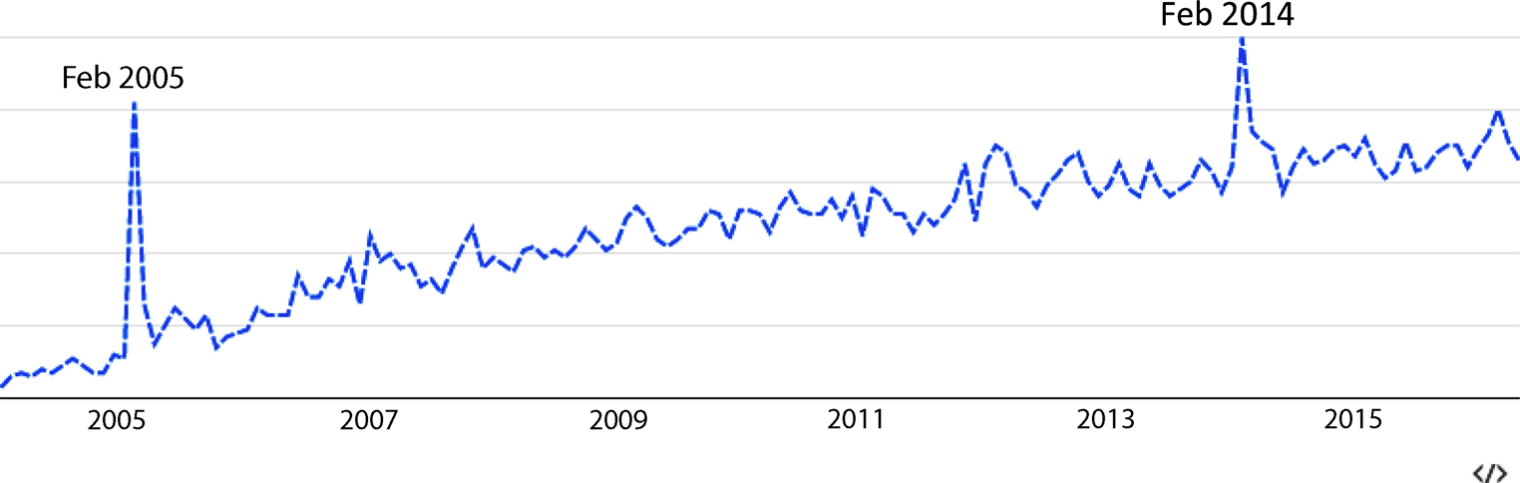
Google search index for ‘Takotsubo cardiomyopathy’ over time

The article by Otten et al. [3] presents a Dutch cohort of STEMI patients from a single large peripheral hospital. Their main finding was that the diagnosis of takotsubo cardiomyopathy in this specific patient cohort seemed to increase during the years, mirroring the amount of publications on PubMed. Nevertheless, the total number of takotsubo patients remained very low (0.4 % in the 2002–2007 period to 0.7 % in the 2008–2013 period). The reason for this increase is most likely a combination of an increased awareness and early patient presentation to the catheterisation lab. While we expect the number of publications to keep on increasing, the total incidence of this disease will most likely remain close to 1 % in this specific patient population. This increased awareness seems to be an important factor, considering the growing number of articles on PubMed, but also represented by the worldwide interest on the Google search engine for takotsubo cardiomyopathy (Fig. 1). We would like to comment on the recommendation to stop ACE inhibitors and continue beta blockade, as suggested by the authors, this recommendation is not supported by a recent large observational cohort [1], where beta blockers did not seem to have any protective effect in the recurrence of takotsubo cardiomyopathy, while patients with ACE inhibitors seemed to have significantly better survival.

A large Polish cohort, published by Zalewska-Adamiec et al. [4], gives us some more insight into the long-term data on hard clinical outcome in takotsubo patients. Although serious cardiac complications (cardiogenic shock, pulmonary oedema, cardiac arrest, and cardiac death) occurred less compared with STEMI patients, this was still seen in almost 15 % of the entire takotsubo cardiomyopathy cohort. Of particular importance is the relatively high number of patients with a cardiac rupture: 3 % compared with 1 % in the STEMI group. This high percentage of cardiac complications is not new since these numbers were also described in a large cohort of takotsubo patients, which was recently published in the New England Journal of Medicine [1]. Therefore, we think we can firmly conclude that the notion that takotsubo cardiomyopathy is a benign disease seems a misconception. These serious complications occur during hospitalisation or shortly afterwards, while in STEMI patients events keep on occurring throughout the first year, a finding fitting both disease entities. Indeed, cardiac death among the takotsubo patients was almost negligible during the two year follow-up period. One limitation of the data is that spasm and spontaneous thrombolysis in the takotsubo group could not be excluded. Furthermore, what is unknown from this publication is what precisely happened in their control group. The amount of patients receiving a primary intervention is unknown and the extent of myocardial damage to the myocardium has not been reported. However, the importance of these unknown factors is irrelevant when we solely focus on the takotsubo patients in this paper (since a real comparable control group for this patient category is intangible).

The final paper by Smeijers and colleagues [5] shifts our focus to psychological distress, illness-related anxiety and personality factors approximately two years after the event. This study uses thorough assessment tools in a small takotsubo patient cohort and compared their findings with heart failure patients and healthy controls. The lesson learned in this study is that patients with takotsubo cardiomyopathy had higher levels of depressive symptoms and illness-related anxiety compared with healthy controls. One of the striking findings is that both depression and anxiety are comparable between takotsubo patients and heart failure patients. Indeed, approximately one-third of takotsubo cardiomyopathy patients and heart failure patients cope with symptoms of depression. This raises the question whether we are overlooking or undertreating these patients?

The authors of this paper conclude that ‘the possibility that the unpredictable nature of takotsubo cardiomyopathy and the lack of a sustained underlying cardiac disease process may result in increased illness-related anxiety and depressive symptoms in TTC patients’. Although this is a valid assumption, we do not know whether or not these symptoms were present in these individuals before the episode of takotsubo cardiomyopathy. a recent report has shown us that more than half the takotsubo patients (55.8 %) had a history or an acute episode of a neurological or psychiatric disorder, conditions that were evident in only 25.7 % of patients with an acute coronary syndrome [1]. The high prevalence of neurological and psychiatric disorders was also reflected by the fact that a substantial number of patients were taking one or more antidepressants (17.1 %).

Besides the abovementioned conclusions, we may also conclude that the increased interest in takotsubo cardiomyopathy has led to many new and long-lasting insights, but thus far not to an easy diagnostic (imaging) biomarker nor treatment. The ESC Heart Failure Association has recently published, for the first time, a position statement, giving a comprehensive review, recommendations and future directions using the current state of knowledge on this intriguing acute heart failure syndrome [9].

The intriguing part is that the number of new questions raised by published articles is increasing at the same pace as our gained knowledge from these data. Is the mechanism in takotsubo cardiomyopathy, for example, a potential clinical cardioprotective mechanism? And if indeed so, can we find this switch to prevent damage also for other diseases?

Fig. 1 illustrates a Google Trends query, demonstrating an increase in worldwide interest over time on Google, the most widely used global search engine. The y‑axis represents the ‘takotsubo cardiomyopathy’ syndrome portion of Google search volume after indexing and normalisation. Peak 1 probably represents the increased attention by several high impact papers in 2005 [6, 7]. Peak 2 in February 2014 probably represents the worldwide news coverage ‘you can die from a broken heart’ based on a JAMA Internal Medicine article by Carey et al. [8].
